# Evidence for the Involvement of Spinal Cord-Inhibitory and Cytokines-Modulatory Mechanisms in the Anti-Hyperalgesic Effect of Hecogenin Acetate, a Steroidal Sapogenin-Acetylated, in Mice

**DOI:** 10.3390/molecules19068303

**Published:** 2014-06-19

**Authors:** Jullyana S.S. Quintans, Rosana S.S. Barreto, Waldecy de Lucca Júnior, Cristiane F. Villarreal, Carla M. Kaneto, Milena B.P. Soares, Alexsandro Branco, Jackson R.G.S. Almeida, Alex G. Taranto, Angelo R. Antoniolli, Rivelilson M. Freitas, Lucindo J. Quintans-Júnior

**Affiliations:** 1 Departamento de Fisiologia. Universidade Federal de Sergipe-UFS, Av. Marechal Rondom, s/n, São Cristóvão, Sergipe, CEP 49.000-100, Brazil; E-Mails: jullyanas@yahoo.com.br (J.S.S.Q.); rosanasfisio@hotmail.com (R.S.S.B.); wlucca1@gmail.com (W.L.J.); aroberto@ufs.br (A.R.A.); 2 Faculdade de Farmácia, Universidade Federal da Bahia, Salvador, Bahia, CEP 40.170-115, Brazil; E-Mail: cfv@conveniado.bahia.fiocruz.br; 3 Centro de Biotecnologia e Terapia Celular, Hospital São Rafael, Salvador, Bahia, CEP 41.253-190, Brazil; E-Mails: carlakenato@bahia.fiocruz.br (C.M.K.); milena@bahia.fiocruz.br (M.B.P.S.); 4 Colegiado de Ciências Farmacêuticas, Universidade Estadual de Feira de Santana, Feira de Santana, Bahia, CEP 44.036-900, Brazil; E-Mail: branco@uefs.br; 5 Colegiado de Ciências Farmacêuticas. Universidade Federal do Vale do São Francisco, Petrolina, CEP 56.304-205, Pernambuco, Brazil; E-Mail: jackson.guedes@univasf.edu.br; 6 Curso de Farmácia, Universidade Federal de São João Del-Rei, Campus Centro Oeste Dona Lindu, Divinópolis, Minas Gerais, CEP 35.501-296, Brazil; E-Mail: proftaranto@hotmail.com; 7 Departamento de Farmácia, Universidade Federal do Piauí, Teresina, Piauí, CEP 64.049-550, Brazil; E-Mail: rivelilson@pq.cnpq.br

**Keywords:** hecogenin acetate, steroidal sapogenin, spinal cord, c-fos, IL-1β, pain

## Abstract

Hecogenin is a steroidal sapogenin largely drawn from the plants of the genus Agave, commonly known as ‘sisal’, and is one of the important precursors used by the pharmaceutical industry for the synthesis of steroid hormones. Hecogenin acetate (HA) is a steroidal sapogenin-acetylated that produces antinociceptive activity. Thus, we evaluate the antihyperalgesic profile of HA in mice in inflammatory models, as well as its possible involvement with c-fos expression on spinal cord area and cytokines to produces analgesic profile. Acute pretreatment with HA (5, 10, or 20 mg/kg; i.p.) inhibited the development of mechanical hyperalgesia induced by carrageenan, TNF-α, dopamine and PGE2. Additionally, the immunofluorescence data demonstrated that acute pretreatment with HA, at all doses tested, significantly inhibited Fos-like expression in the spinal cord dorsal horn normally observed after carrageenan-inflammation. Moreover, HA did not affect the motor performance of the mice as tested in the Rota rod test. This antinociceptive profile seems to be related, at least in part, to a reduction of pro-inflammatory cytokines, as IL-1β. The present results suggest that HA attenuates mechanical hyperalgesia by blocking the neural transmission of pain at the spinal cord levels and by cytokines-inhibitory mechanisms.

## 1. Introduction

Pain management remains one of the greatest challenges of contemporary medicine, nevertheless, there are a relatively limited list of treatment options for pain [1]. Although a considerable number of analgesic drugs are available for the treatment of painful disorders, the search for development of new compounds as therapeutic alternatives continues since the available analgesic drugs exert a wide range of side effects [2,3]. In this regard, opioids and nonsteroidal anti-inflammatory drugs (NSAIDs) have been the main stay of pain treatment. Presently, there is concern that success in the development of new analgesic agents is limited [1].

Although the expansion of synthetic medicinal chemistry in the last decades caused the proportion of new drugs based on natural products to drop to ~50%, 13 natural product–derived drugs were approved in the U.S. Food and Drug Administration (FDA) between 2005 and 2007, with five of them being the first members of new classes [4]. For the treatment of pain the latest example of the natural product approved by FDA is Ziconotide (Prialt^®^), a peptide toxin obtained from Conus magus (marine gastropod mollusk in the family Conidae), and it is the first member in the new drug class of selective N-type voltage-sensitive calcium-channel blockers [5]. Currently, because of their relatively low cost and easy availability in several countries (mainly developing countries), natural products could be used as synthetic models of more selective and powerful drugs [6,7,8].

Hecogenin is a steroidal sapogenin largely drawn from the plants of the genus *Agave* (commonly known as ‘sisal’), and belongs to family Agavaceae, widely distributed in tropical and subtropical regions throughout the world [9]. ‘Sisal’ species possess both commercial importance (as source of industrial fibers) and medicinal importance and they have been used in Chinese medicine for the treatment of scabies and tumors, as well as for painful and inflammatory conditions [10,11]. The steroidal sapogenins obtained from ‘sisal’ species (mainly diosgenin and hecogenin) are important precursors used by the pharmaceutical industry for the synthesis of steroid hormones, such as adrenal cortical hormones (cortisone, cortisol, prednisolone, prednisone, dexamethasone, betamethasone, triamcinolone, *etc.*), sexual hormones (progesterone), and protein anabolic hormones (stanozolol, methandienone) [12,13,14]. However, there is little scientific information about the biological properties of hecogenin in painful and inflammatory conditions. Peana *et al.* [15] demonstrated hecogenin reduced paw edema induced by carrageenin, but also produced gastric mucous lesions in higher doses. Cerqueira *et al.* [16] proposed that the anti-inflammatory property of hecogenin was produced by COX-2 inhibition. Conversely, theses authors demonstrated that pretreatment with hecogenin produced significant gastroprotective profile mediated by K+ATP channels. 

**Figure 1:**
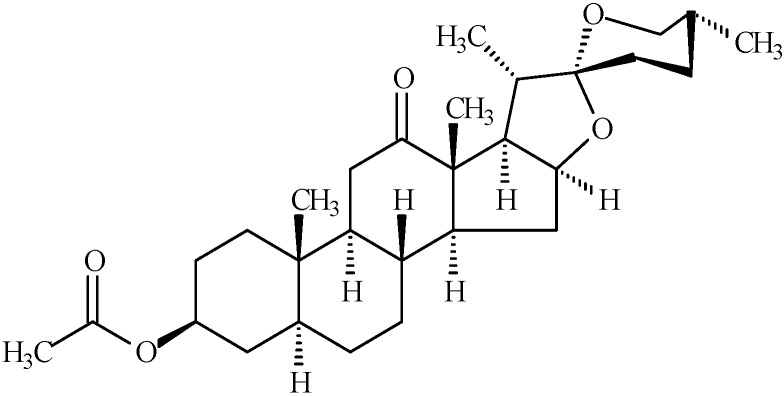
Chemical structure of hecogenin acetate.

Recently, our group reported that hecogenin acetate (HA) (Figure 1), a steroidal sapogenin-acetylated, possesses antinociceptive activity using the tail-flick test and suggested the possible involvement of descending pain-inhibitory mechanisms [17]. Now, the antinociceptive effect of HA was investigated in inflammatory hyperalgesia models on mice, examining HA-evoked cFos immunoreactivity in spinal cords levels and cytokines enhancement by RT-PCR technique. We hypothesized that HA may be acting by spinal cord inhibitory mechanisms to produces anti-hyperalgesic profile and that the reduction of proinflammatory cytokines may contribute to this analgesic effect.

## 2. Results and Discussion

The current study shows that systemic administration of hecogenin acetate (HA) could able to reduced inflammatory hyperalgesia in a dose-dependent manner and its reduce fos expression in the dorsal horn of the spinal cord normally produced by carrageenan suggesting it reduces central excitability.

**Figure 2:**
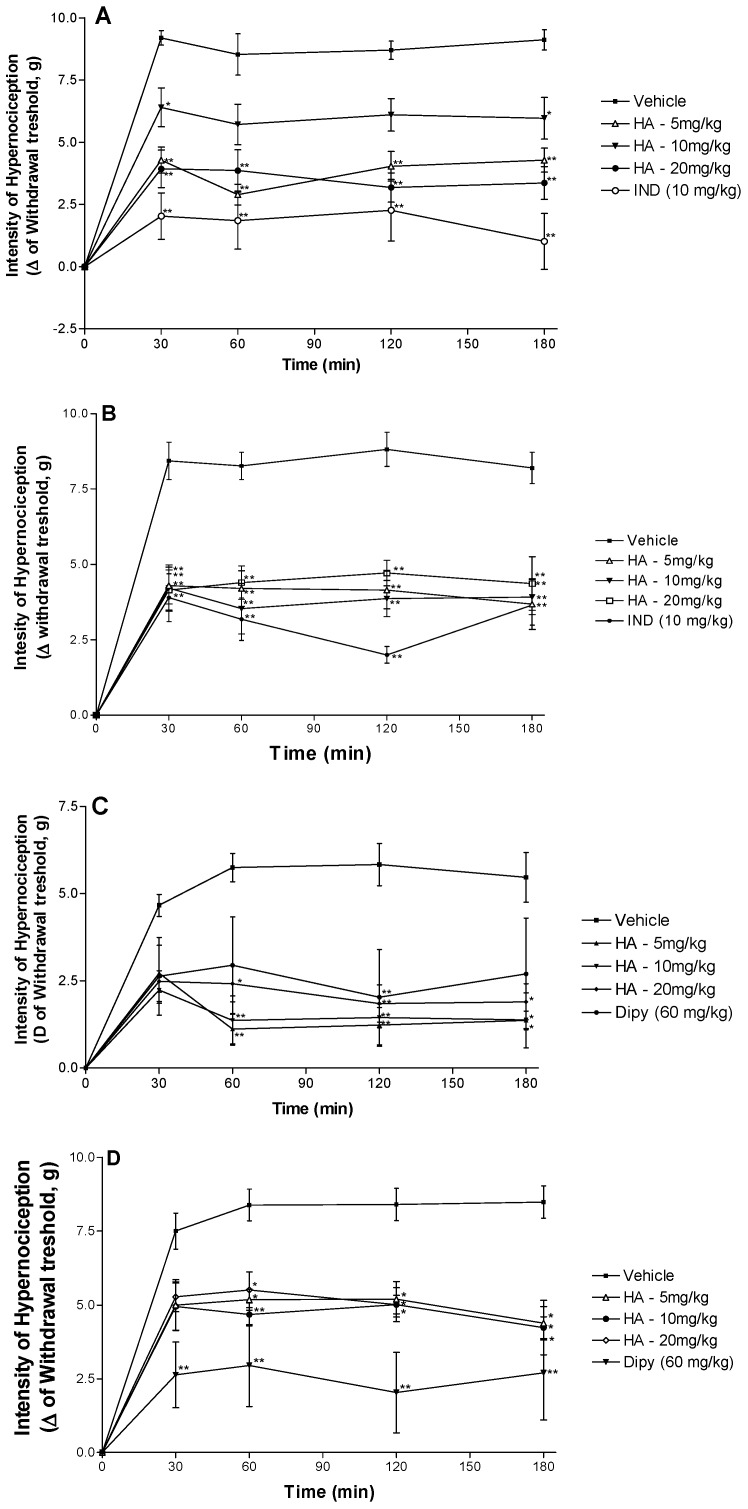
Effect of the acute administration of vehicle, hecogenin acetate (HA, 5, 10 or 20 mg/kg; i.p.) or reference drugs (indomethacin—10 mg/kg; i.p. or dipyrone—60 mg/kg, i.p.) on mechanical hyperalgesia induced by carrageenan (**A**), TNF-α (**B**), dopamine (**C**) or PGE2 (**D**). Each point represents the mean ± SEM of the paw withdrawal threshold (in grams) to tactile stimulation of the ipsilateral hind paw. * *p* < 0.05, ** *p* < 0.01 and *** *p* < 0.001 *versus* control group (ANOVA followed by Tukey test).

As showed in Figure 2, intraplantar injection of carrageenan (300 µg/paw) induced a significant increase in the number of responses to 0.6 g force applied to the inflamed hindpaw when compared to baseline. The increased number of responses was maintained from 30 to 180 min after the carrageenan administration. The systemic administration of HA (5, 10 or 20 mg/kg, i.p.) produced an anti-hyeralgesic effect in this model. Specifically mice treated with HA, all doses, 30 min before carrageenan administration exhibited a significant reduction in the number of responses to repeated application of the 0.6 g mechanical force at all evaluated times, when compared with animals vehicle-treated (control group). As expected, the reference drug (indomethacin, 10 mg/kg, i.p.) showed reductions in the number of responses to the 0.6 g force (Figure 2A). The group of animals that received saline in the sub plantar region, instead of carrageenan, did not present any alteration on the threshold of sensitivity towards the mechanical stimuli (data not shown).

The inhibitory effect of HA (5, 10 or 20 mg/kg, i.p.) on the mechanical hyperalgesia induced by TNF-α is shown in Figure 2B. Hecogenin acetate (5, 10 or 20 mg/kg) was able to reduce mechanical hyperalgesia induced by TNF-α, when compared with animals of the control group (vehicle-treated), similarly to the reference drug (indomethacin, 10 mg/kg, i.p.). Figure 2C shows the inhibitory effect of HA on the mechanical hyperalgesia induced by dopamine. HA, in a higher dose, was able to reduce mechanical hyperalgesia induced by dopamine, when compared with animals of the vehicle group. Intraplantar administration of PGE2 induced a marked mechanical hypersensitivity that was significantly reduced by dipyrone (60 mg/kg, i.p.) and by hecogenin acetate at 5–20 mg/kg, as shown in Figure 2D. The analgesic potency of hecogenin acetate attributed to its ability to inhibit pro-inflammatory cytokines production and seems to be comparable to dipyrone and indomethacin at higher doses. However, future studies should further evaluate the potency of hecogenin acetate compared to other drugs reference.

Hyperalgesia induced by i.pl. injection of carrageenan is widely used for evaluating new anti-hyperalgesic drugs in rodents [18]. In experiments using mice, injection of carrageenan into the plantar surface of animal hind paws produced inflammation and hyperalgesia with similar temporal profile [19]. In this model, there is the occurrence of non-immunereaction, involving inflammatory mediators, including arachidonic acid products (PGE2), mast cells products (histamine, 5-HT), neuropeptides, cytokines (IL-1β and TNFα), NO, nerve growth factor (NGF), leukotrienes B4 (LTB4) and transcription factors (NF-κB) [20,21]. According to Cunha *et al.* [22], this cascade of signalization leads to the release of prostanoids and sympathomimetic amines. Therefore, those final inflammatory mediators can activate peripheral Aδ and C fiber sensory nerve terminals, thus causing the release of substance P and neurokinin A, leading to increases in local blood flow and vascular permeability [23].

Cytokines produced after i.pl. injection of carrageenan may exert in direct cytotoxic effects by the release of NO, reactive oxygen species, eicosanoids and excitatory amino acids (EAA - glutamate and aspartate) EAA [24]. Alternatively, IL-1β induced by carrageenan might sensitize spinal neurons through the induction of nociceptive neuropeptides expression, such as substance P (SP) and nerve growth factor (NGF). It also has been shown that nociceptive stimulus induced by glutamate, PGs, histamine or 5-HT carrageenan-induced release resulted on direct nociceptors sensitization, culminating with simultaneous thermal and mechanical hyperalgesia [25]. We showed that acute HA administration, all doses, was able to reverse the hyperalgesic response induced by carrageenan, thus suggesting a possible inhibitory effect on the inflammatory cascade.

It is well know that cytokine TNF-α is recognized as a potent pro-inflammatory endogenous substance, which is rapidly produced in large quantities by macrophages in response to inflammatory stimuli such as bacterial infection [26]. TNF-α interacts with target cells through high-affinity membrane receptors, as tumor necrosis factor receptor type 1 (TNFR1 or p55) and type 2 (TNFR2 or p75) [27]. Thus, TNF-α-induced hyperalgesia is mediated by prostanoids and sympathetic amines. We demonstrated that HA was able to reverse the hyperalgesic response induced by TNF-α, thus suggesting a possible inhibitory effect on the inflammatory cascade, as observed with the treatment with indomethacin.

Taking those findings into account, we evaluated whether the antinociceptive activity of hecogenin acetate involves the blockade of sensitization or activation of the nociceptor through evaluation of its effect on hyperalgesia induced by PGE2 and dopamine [18]. As the hyperalgesia induced by these mediators is independent of the final production of other inflammatory mediators or recruitment of cells, such as neutrophils [28] and as it has been demonstrated that HA administration was able to maintain the baseline nociceptive threshold, we can then suggest the possibility that HA interacts with dopamine or EP receptors; consequently, the paths of its analgesic effects may be through the action not only at the inflammatory level but also from a possible involvement of neuronal pathways [29], as suggested by Brito *et al.* [29] and Gama *et al.* [30]. 

**Figure 3:**
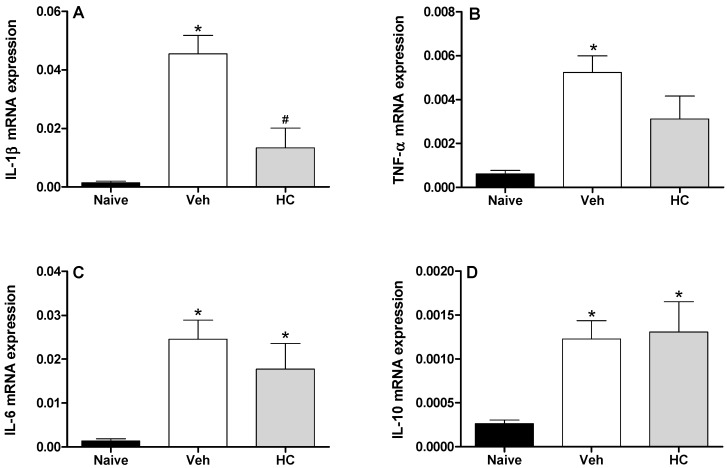
Effect of treatment with hecogenin acetate (HA) on IL-1β mRNA (**A**), TNF-α mRNA (**B**), IL-6 mRNA (**C**), and IL-10 mRNA (**D**) paws levels. Mice were injected with HA (20 mg/kg) or vehicle (Veh; XX; control group) by the intraperitoneal route 30 min before the intraplantar injection of complete Freund’s adjuvant (CFA). The paw levels of cytokines were measured by Real Time PCR 2 h after the CFA injection. Data are reported as means ± SEM; *n* = 6 mice per group. * *p* < 0.05 *vs.* naive group; # *p* < 0.05 *vs.* vehicle-treated group, two-way ANOVA followed by Tukey’s test.

**Figure 4:**
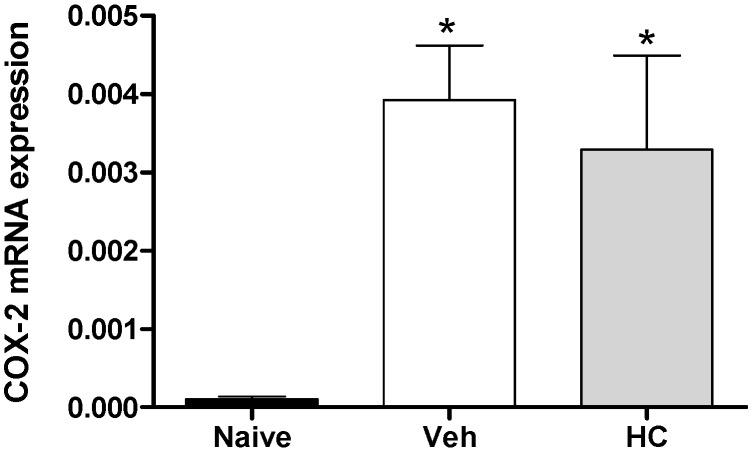
Effect of treatment with hecogenin on COX-2 mRNA paw levels. Mice were injected with hecogenin (HC; 20 mg/kg) or vehicle (Veh; control group) by the intraperitoneal route 30 min before the intraplantar injection of complete Freund’s adjuvant (CFA). The paw levels of COX-2 mRNA were measured by Real Time PCR 2 h after the CFA injection. Data are reported as means ± SEM; *n* = 6 mice per group. * *p* < 0.05 *vs.* naive group, one-way ANOVA followed by Tukey’s test.

In fact, the acute pretreatment with hecogenin acetate increased threshold sensitivity towards mechanical stimuli in carrageenan-, TNF-α, PGE2- or dopamine-induced mechanical hyperalgesia, this compound is suggested to have produced an inhibition of the inflammatory cascade. Previously, Peana *et al.* [15] and Cerqueira *et al.* [16] have shown the antiedematogenic and anti-inflammatory effects of hecogenin, and they demonstrated that anti-inflammatory activity of hecogenin may be involvement inhibitory of cytokines pathway, at least partially. This hypothesis has been confirmed by our results of Real Time PCR analysis when we demonstrated that HA treatment could inhibit the expression of IL-1β (Figure 3). However, acute treatment with HA did not alter TNF-α (Figure 3B), and IL-6 (Figure 3C); and the expression of the anti-inflammatory cytokine IL-10 (Figure 3D). In addition, Cerqueira *et al.* [16] proposed that hecogenin, as non-acetylated, increased COX-2 expression in ethanol-induced gastric ulcer in rodents and who also suggest that hecogenin produces beneficial effect in gastric injury through mechanisms involving the inhibition of inflammatory cell infiltration and lipid peroxidation, up-regulation of the COX-2/PG pathway and K+ATP channels. Differently, we demonstrated that acute treatment with HA was not able to change COX-2 expression, by assessed mRNA COX-2 levels (Figure 4).

As it is well known, peripheral or central opioid receptor activation could lead to the decrease of the pain sensation towards an inflammatory stimulus, through the NO-cGMP-K+ATP channel pathway activation [31]. Based on both suggestions that HA promotes regulation of K+ATP channels and the inhibitory effect of opioids on the spinal nociceptive transmission, and as our group has recently demonstrated, for the first time, that systemic administration of HA, at doses that did not induce motor performance alterations, produced consistent antinociceptive effect, probably mediated by opioid system and descending pain-inhibitory pathways activation [30], we investigated whether analgesic-like profile of HA was due to the involvement of spinal cord dorsal horn pathways through immunohistochemical approach.

**Figure 5:**
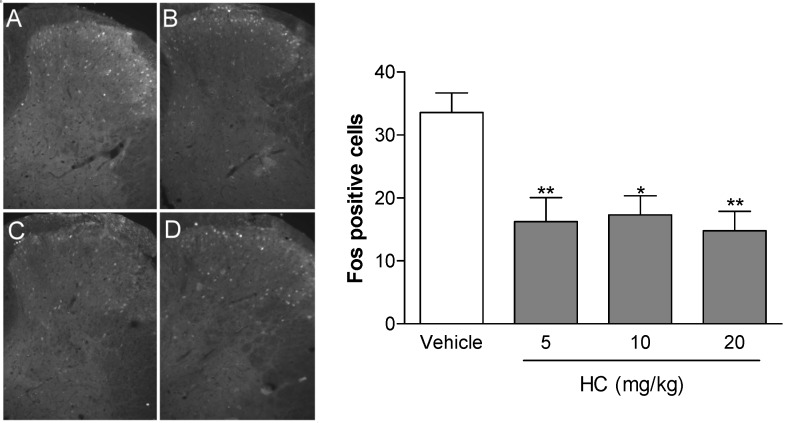
Immunofluorescence for Fos protein in the spinal cord dorsal horn, Ninety minutes after the intraperitoneal injection of vehicle (**A**) hecogenin acetate at doses of 5 (**B**), 10 (**C**) and 20 (**D**) mg/Kg. The bar graph shows (below and right side) average Fos positive cells compared with the vehicle-treated group (white bar) Values represent mean ± S.E.M. (*n* = 6, per group). * *p* < 0.05, ** *p* < 0.01 *vs.* control (one-way ANOVA followed by Tukey’s test).

Ninety minutes after the intraperitoneal injection of HA, the average number of neurons showing Fos protein in the spinal cord dorsal horn was significantly (*p* < 0.05) reduced (Figure 5) at doses of 5, 10 and 20 mg/Kg when compared with control (Vehicle).

The superficial dorsal horn of the spinal cord, particularly substantia gelatinosa (SG), is a major projection site of small-diameter afferent nerve fibers that predominantly transmit nociceptive signals. SG neurons also receive descending inputs from the brainstem [32]. Baba *et al.* [33] demonstrated using an *in vitro* spinal cord slice preparation that peripheral inflammation can facilitate A-beta fiber-mediated synaptic inputs to dorsal horn of the spinal cord, mainly SG, and also produce an increase in c-fos expression.

The protooncogene c-fos, when activated, makes the immunologically detectable nuclear protein Fos [34]. A striking attribute of Fos is that it is rapidly expressed in central neurons after noxious stimuli [35]. Previous studies demonstrated that the increased c-fos expression is a transient reaction of spinal neurons in painful conditions, as in chronic pain [35,36]. The causes of that spinal cord nociceptive neuronal hyperactivity during certain types of pain remain unclear. It may derive from the constant barrage of peripheral input [37], but may also reflect impairments of descending modulation, as previously showed by HA [30]. Anyway, the value of the analysis of Fos expression to monitor the nociceptive activity of large neuronal populations is solid [35].

In addition, CNS-depressant drugs, such as gabapentin, frequently used as analgesic in some types of painful conditions, inhibit glutamatergic excitatory neurotransmission at the spinal dorsal horn and decrease Fos expression when they produce analgesic effect [38]. Thus, our results suggests that the inhibition of neuronal hyperactivity at the spinal cord dorsal horn (by a significantly decrease of Fos expression) could account for the analgesic efficacy of hecogenin acetate, in all doses tested, in carrageenan-induced inflammatory nociception. Hence, it may also decrease neuronal activation at the spinal cord horn, by depressing descending facilitation through periaqueductal gray (PAG) activation, as previously demonstrated by our group [30].

Earlier studies suggested that the CNS depression and the non-specific muscle relaxation effects can reduce the response of motor coordination and might invalidate the behavioral test results, including mechanical hyperalgesic tests [28,39]. Since Brito *et al.* [29] showed the anxiolytic and antidepressant effects of hecogenin and as these activities can induce impair in motor coordination, we assessed that acute treatment with HA, at the doses tested, did not have any performance alteration in the rota-rod apparatus (data no shown).

## 3. Experimental

### 3.1. Animals

All experimental protocols were approved by the Animal Care and Use Committee (CEPA/UFS # 04/12) at the Federal University of Sergipe, and handling procedures were in accordance with the Guide for the Care and Use of Laboratory Animals (NIH - National Institutes of Health) for the use of animals in pain research [40]. Male *Swiss* mice (32–39 g), 2–3 months of age, were used throughout this study. The animals were randomly housed in appropriate cages at 21 ± 2 °C on a 12 h light/dark cycle (lights on from 6:00 a.m. to 6:00 p.m.) with free access to food (Purina^®^, Brazil) and water. Before the experiments, the animals were acclimatized to the laboratory for at least 1h. Mice were used only once in each test. Experiments were carried out between 9:00 a.m. and 2:00 p.m. in a quiet room. All experiments involving the behavioral analysis were carried out by the same visual observer and in a double-blind manner.

### 3.2. Hyperalgesic Stimulus and Nociceptive Threshold Evaluation

Mechanical hyperalgesia was tested in mice as reported by Cunha *et al.* [41], with alterations as previously published [39]. In a quiet room, mice were placed in acrylic cages (12 × 10 × 17 cm) with wire-grid floors 15–30 min before starting the test. This method consisted of evoking a hindpaw flexion reflex with a hand-held force transducer (electronic anesthesiometer, Model EFF 302, Insight^®^, Ribeirão Preto-SP, Brazil) adapted with a polypropylene tip. The investigator was trained to apply the tip perpendicularly to the central area of the hindpaw with a gradual increase in pressure. The end point was characterized by the paw withdrawal followed by clear flinching movements. After this response, the intensity of the pressure was automatically recorded. The intensity of stimulus was obtained by averaging four measurements performed with minimal intervals of 3 min. The animals were tested before and after treatments.

Mice were divided into five groups (*n* = 8, per group), which were treated with vehicle (saline + tween 80, 0.2 mL for 1 mL - saline; i.p.), hecogenin acetate (HA, 5, 10 or 20 mg/kg; i.p.), indomethacin (10 mg/kg; i.p.) or dipyrone (60 mg/kg; i.p.). Thirty min after treatment, 20 μL of carrageenan (300 µg/paw), PGE2 (100 ng/paw), DA (30 μg/paw) or TNF-α (100 pg/paw) were injected subcutaneously into the subplantar region of the hindpaw, as described by Cunha *et al.* [41] and Villarreal *et al.* [18]. The degree of hyperalgesia was evaluated at 0.5, 1, 2, and 3 h after the injection of hyperalgesic agents.

### 3.3. Motor Function Assay (Rota-Rod Test)

To evaluate the possible non-specific muscle-relaxant or sedative effects of hecogenin acetate in the doses used, mice were submitted to the rota-rod test (AVS^®^, Brazil) according to Quintans-Junior *et al*. [42]. The animals were selected 24 h previously by eliminating those mice which did not remain on the bar for two consecutive periods of 240 s. Mice were pre-treated with diazepam (DZP, 3 mg/kg, i.p., reference drug), HA (5, 10 or 20 mg/kg, i.p.) or vehicle (saline + Tween 80, 0.2 mL for 1 mL-saline, i.p.) and 1 h later were placed on a rotating rod. The latency to falling was measured up to 180 s. The results are expressed as the average time (s) during which the animals remained on the rota-rod apparatus in each group.

### 3.4. Immunofluorescence

Ninety minutes after the intraperitoneal injection of HA at doses of 5, 10 and 20 mg/Kg or vehicle (Saline + Tween 80, 0.2 mL for 1 mL-saline), the animals (*n* = 6, per group) were perfused with phosphate buffer (0.01 M) saline isotonic (PBS) followed by 10% buffered formalin (100 mM). The spinal cord (L4-L6) were removed and stored at −80 °C for the imunofluorescence against Fos protein.

The protocol for immunofluorescence was based on prior [43,44]. Frozen serial transverse sections (20 µm) of the whole spinal cord were collected on gelatinized glass slides. After washing with PBS, the slices were incubated with 0.1 M glycine in PBS for 10 min. Non-specific protein binding was blocked by the incubation of the sections for 30 min in a solution containing 2% BSA. Then, the sections were incubated overnight with rabbit anti-Fos as primary antibodies (1:2000). Afterwards, the sections were incubated for 2 hr with donkey anti-rabbit IgG -Alexa Fluor 594 (1:2000). Sections were cover slipped with Fluoromount G. As a control for non-specific labeling, sections were incubated without primary antibody. After each stage, slides were washed with PBS five times for 5 min.

### 3.5. Acquisition and Analyses of Images

Light level photomicrographs of spinal cord sections were acquired for each animal with an Axioskop 2 plus, Carl Zeiss^®^, Germany. Neurons were counted by the free software Image J (National Institute of Health) using a plug-in (written by the authors) that uses the same level of label intensity to select and count the Fos-positive cells.

### 3.6. Real Time PCR

Mice were slightly anesthetized with halothane and received 10 µL of complete Freund’s adjuvant (CFA; 1 mg/mL of heat-killed Mycobacterium tuberculosis in 85% paraffin oil and 15% mannide monoleate; Sigma, St. Louis, MO, USA) subcutaneously in the plantar region of the right hind paw, according to a method reported previously [45]. The transcription of TNF-α (tumor necrosis factor alpha), IL-1β (Interleukin 1 beta), IL-6 (Interleukin 6), IL-10 (Interleukin 10) and COX-2 (cyclooxygenase type 2) genes was evaluated by real-time quantitative polymerase chain reaction (qRT-PCR) in mice sacrificed two hours after the CFA injection. Total RNA was isolated from the paw tissue with TRIzol reagent (Invitrogen Corporation, Carlsbad, California, CA, USA), and the concentration was determined by photometric measurement. High Capacity cDNA Reverse Transcription Kit (Applied Biosystems, Foster City, CA, USA) was used to synthesize cDNA from 1 μg of RNA following the manufacturer’s recommendations. qRT-PCR assays were performed to detect the expression levels of IL-1β, TNF-α, IL-6, IL-10 and COX-2 genes. Amplification mixtures for qRT-PCR contained 20 ng template cDNA, 10 μL Taqman Master Mix (Applied Biosystems) and probes in a final volume of 20 μL. All reactions were run in duplicate on an ABI7500 Sequence Detection System (Applied Biosystems) under standard thermal cycling conditions. Experiments with coefficients of variation greater than 5% were excluded. A no-template control (NTC) and no-reverse transcription controls (No-RT) were also included. The results are presented as the fold-increase of expression of the individual mRNAs, with the target internal control GADPH using the cycle threshold method. 

### 3.7. Drugs and Reagents

λ-Carrageenan, tumor necrosis factor-alpha (TNF-α), prostaglandin E2 (PGE2), dopamine (DA), ethylenediamine tetraacetic acid (EDTA), Tween 80, fluoromount G, glycine and bovine serum albumin (BSA) and hecogenin acetate (HA, ~90% purity) were purchased from Sigma (Sigma, St. Louis, MO, USA). Indomethacin and dipyrone were purchased from União Quimica (Brazil). c-Fos Antibody, a rabbit polyclonal IgG, was obtained from Santa Cruz Biotechnology (Dallas, Texas, TX, USA) and Alexa Fluor^®^ 488, a Donkey Anti-Rabbit IgG (H+L), was obtained from Life technology (Grand Island, New York, NY, USA).

### 3.8. Statistical Analysis

Data are presented as means ± standard error of the mean (SEM) of measurements made on 6–8 animals in each group. Comparisons between three or more treatments were made using two-way analysis of variance (ANOVA) followed by Tukey’s test. In all cases, differences were considered significant if *p* < 0.05. All statistical analyses were carried out using Graph Pad Prism 5.0 (Graph Pad Prism Software Inc., San Diego, CA, USA).

## 4. Conclusions

Together, our results clearly indicate that HA displays significant anti-hyperalgesic effect in animal protocols. The precise mechanism by which HA promotes its effects is not clear, but the compound’s ability to modulate spinal cord dorsal horn and inhibit IL-1β expression appear to be likely one of the possible mechanisms. However, it is possible that another central or peripheral mechanism, not studied in present work, may be related to the analgesic effect of hecogenin acetate. Therefore, this steroidal sapogenin may be of potential interest in the development of new clinically relevant drugs for the management of painful conditions.
